# Mechanical Disruption of Tumors by Iron Particles and Magnetic Field Application Results in Increased Anti-Tumor Immune Responses

**DOI:** 10.1371/journal.pone.0048049

**Published:** 2012-10-25

**Authors:** Myriam N. Bouchlaka, Gail D. Sckisel, Danice Wilkins, Emanual Maverakis, Arta M. Monjazeb, Maxwell Fung, Lisbeth Welniak, Doug Redelman, Alan Fuchs, Cahit A. Evrensel, William J. Murphy

**Affiliations:** 1 Department of Microbiology and Immunology, University of Nevada School of Medicine, Reno, Nevada, United States of America; 2 Department of Dermatology, University of California Davis, Sacramento, California, United States of America; 3 VA Northern California Health Care System, Sacramento, California, United States of America; 4 Department of Radiation Oncology, University of California Davis, Sacramento, California, United States of America; 5 Departments of Dermatology and Pathology, University of California Davis, Sacramento, California, United States of America; 6 Department of Physiology and Cell Biology, University of Nevada, Reno, Nevada, United States of America; 7 Department of Dermatology and Internal Medicine, University of California Davis, Sacramento, California, United States of America; 8 Department of Chemical Engineering, University of Nevada, Reno, Nevada, United States of America; 9 Departments of Biomedical and Mechanical Engineering, University of Nevada, Reno, Nevada, United States of America; 10 Graduate Program in Biomedical Engineering, University of Nevada, Reno, Nevada, United States of America; University of Nebraska Medical Center, United States of America

## Abstract

The primary tumor represents a potential source of antigens for priming immune responses for disseminated disease. Current means of debulking tumors involves the use of cytoreductive conditioning that impairs immune cells or removal by surgery. We hypothesized that activation of the immune system could occur through the localized release of tumor antigens and induction of tumor death due to physical disruption of tumor architecture and destruction of the primary tumor *in situ*. This was accomplished by intratumor injection of magneto-rheological fluid (MRF) consisting of iron microparticles, in Balb/c mice bearing orthotopic 4T1 breast cancer, followed by local application of a magnetic field resulting in immediate coalescence of the particles, tumor cell death, slower growth of primary tumors as well as decreased tumor progression in distant sites and metastatic spread. This treatment was associated with increased activation of DCs in the draining lymph nodes and recruitment of both DCs and CD8(+)T cells to the tumor. The particles remained within the tumor and no toxicities were observed. The immune induction observed was significantly greater compared to cryoablation. Further anti-tumor effects were observed when MRF/magnet therapy was combined with systemic low dose immunotherapy. Thus, mechanical disruption of the primary tumor with MRF/magnetic field application represents a novel means to induce systemic immune activation in cancer.

## Introduction

The greatest challenge in the treatment of cancer is the control and eradication of metastatic disease. Conventional cancer treatments, although efficient at debulking tumor, elicit poor systemic anti-tumor effects presumably due to both loss of the tumor antigen pool (surgery) and/or cytoreductive therapies (e.g., chemotherapy and radiation) which can also attack immune effector cells. There are minimally invasive therapies targeting focal cancer by means of cryosurgery and external beam radiotherapy [1]–[3]. These alternative modalities have shown promise in a number of cancers including colon and breast cancer [2], [4]–[10]. However, immune cell suppression within the tumor following such therapies may limit the induction and amplification of an immune response. We hypothesized that a novel means to induce *in situ* tumor destruction through mechanical means offers advantages in that it provides an antigen source for DCs and activate local inflammatory pathways resulting in greater DC activation.

We hypothesize that mechanical disruption of the tumor *in situ* augments immune responses due to induction of tumor death and subsequent antigen release. Iron microparticles were injected as a magneto-rheological fluid (MRF) into orthotopic primary tumors in which the particles are dispersed randomly with the tumor. Following application of a local magnetic field there was immediate coalescence due to the particles aligning along the lines of the magnetic flux, resulting in the disruption of tumor architecture and tumor necrosis. In other methods, superparamagnetic iron oxide (SPIO) have been used to induce hyperthermia by means of altering electromagnetic fields [11]. In the current methodology, permanent magnets were used. Permanent magnets do not depend on external electric field but have a residual magnetism as opposed to electromagnets that generate magnetism by application of electric fields. This application and switching of electric fields results in heat induction, which is not the case with the permanent magnets [12], [13]. Tumors treated with daily application of magnetic field showed increased necrotic cell death and recruitment of activated DCs to the tumor-draining-LNs (DLNs). This resulted in increased systemic anti-tumor immunity and increased antigen-specific CD8(+) T cells homing to treated-primary tumors. Finally, the anti-tumor effects induced by MRF/magnetic field application could be further augmented in conjunction with immunotherapy. These data suggest that primary tumor disruption and death induced by MRF and magnetic field application can result in induction of disseminated anti-tumor responses.

## Results

### MRF and Magnet Application *in vivo* Results in Tumor Death

**Figure 1:**
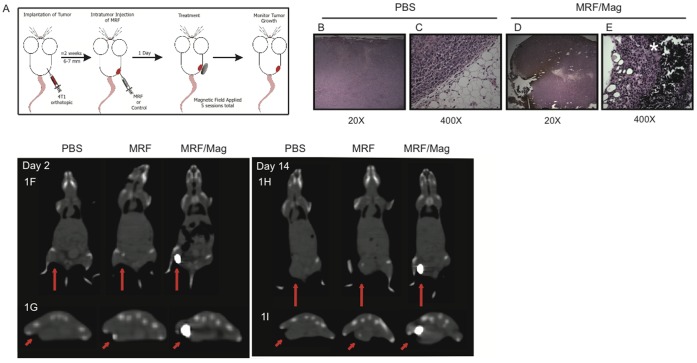
Histological assessment and biodistribution of iron particles following intratumoral MRF/magnetic field application. (A) Experimental design and schedule of treatment: 4T1 cells were injected s.c into the mammary fat pad of female BALB/c mice. When tumors reached 6–7 mm, 100 µL of 60% MRF w/v in PBS was injected into the tumor (i.t) for treatment groups or 100 µL PBS in control group. Iron particles are dispersed randomly in the tumor. One group received magnetic field treatments by direct application of permanent magnets on the primary tumor. Mice received 5 min/session of magnet treatments using a 0.4 Tesla magnet starting 24 hours after MRF injection for 5 consecutive days. Another group received MRF i.t and no further treatments. During magnetic field treatment, the iron particles aggregate and lead to (B–E) At the end of MRF/magnet treatment, tumors were collected, fixed and sectioned in formalin and then stained with H&E. (B–C) Images of PBS or (D–E) MRF/magnet treated groups (20X or 400X magnification). Iron particles depicted by the brownish particles (1D) or black particles (white star) (1E). One representative experiment of three independent experiments. (n = 3 mice/group). (F–I) Trafficking of iron particles to distant sites by *in vivo* imaging 2 days (F–G) and 14 days (H–I) post iron injections as depicted by CT-scan images of one representative animal per group. Groups include PBS, MRF and MRF/magnet treatment showing sagittal CT-scanning (F, H) and coronal CT-scanning of the lower hind area where tumor was inoculated (G, I). Red arrows point at tumor and site of iron particles (white area). n = 3 mice/group from one experiment.

We utilized a mouse mammary breast carcinoma model that highly mimics human breast cancer [14], [15]to investigate the efficacy of magneto-rheological fluid (MRF) and magnetic treatments in the induction of mechanical destruction of the tumor *in situ* and anti-tumor immune responses. Primary orthotopic tumors of the metastatic murine breast cancer cell line 4T1 [15]–[17] were established by injecting cells into the mammary fat pad of female BALB/c mice. Two weeks after tumor inoculation, 100 µL MRF consisting of 6–8 micron-size iron oxide particles in a 60% by weight suspension with PBS was injected directly into the tumor (Fig. 1A). The particles were dispersed within the tumor (Fig. 1D–E) and remained localized within the tumor throughout the study. Control recipients received intratumor (i.t) injections of PBS (Fig. 1B–C) or MRF alone with no magnetic field application. The third group received MRF followed by daily application of a permanent magnet over the primary tumor for a total of 5 min/session for 5 consecutive days (Fig. 1A). No heat is generated using this approach. The dosing and percentage of iron in MRF in suspension as well as duration and frequency of magnetic field application were evaluated in pilot studies. We have tested MRF at 40%, 60% and 80% w/v as well as the duration of magnetic field application for 1 min, 5 min or 10 min a session for only 1 day, every other day or daily for 5 consecutive days. Different strength of magnetic field was not tested. From the preliminary experiments (data no shown), it was determined that the regimen presented in Figure 1A was optimal for immune activation and anti-tumor effects. The maintenance of MRF at the site of injection was discernible by the persistence of magnetic attraction to the MRF in the tumor during the magnet treatments. Injection at the tumor site with MRF followed by magnetic field application results in an immediate consolidation of the particles, which were initially dispersed into the tumor as determined by histological examination of the primary tumor (Fig. 1B–E). In addition, the MRF stayed localized and coalesced within the primary tumor after magnetic field application as determined by CT-scan photomicrographs (Fig. 1F–G). The iron particles did not disperse to distant sites including the abdomen, lung and brain of the mice in groups that received i.t injections of MRF alone or with magnet treatments as compared to PBS–treated animal (Fig. 1F and 1H and data not shown). The iron particles remained at the primary tumor for at least 14 days after MRF injections (Fig. 1H–I) and no long-term toxicity has been observed with the magnetic field treatments.

### Slower Primary Tumor Progression and Inhibition of Bone Metastasis as a Consequence of Death of Primary Tumor following MRF and Magnetic Field Application

**Figure 2:**
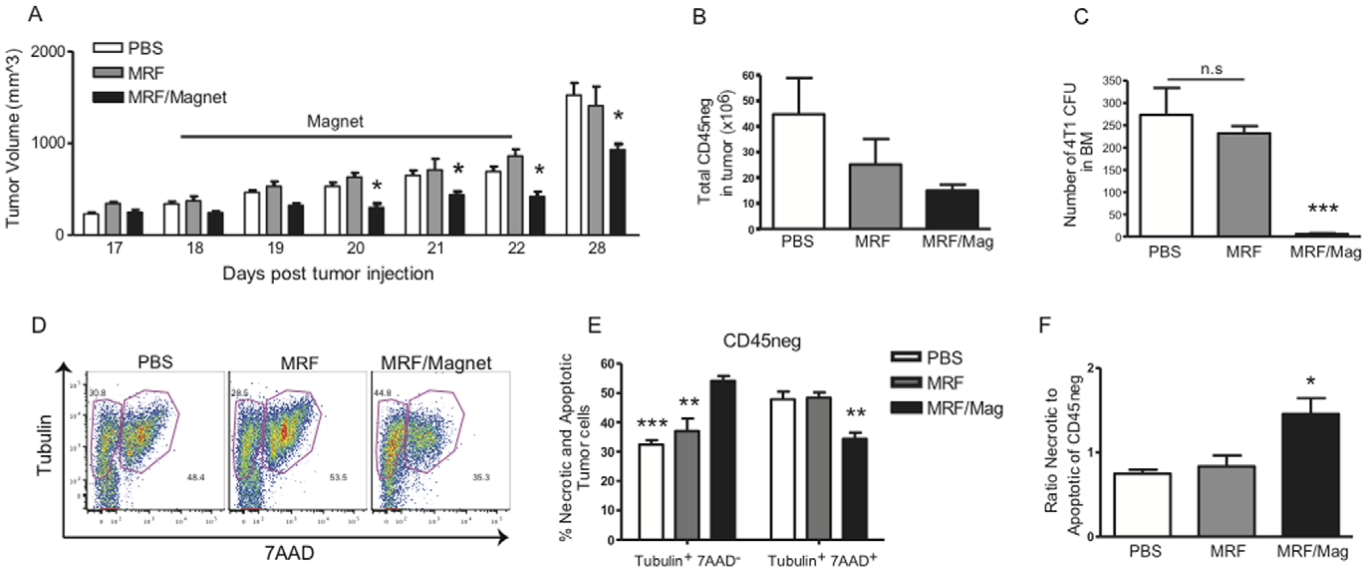
Inhibition of local and systemic tumor growth as a result of increased primary tumor necrosis after MRF implantation and magnetic field treatment. 4T1 tumors were established into the mammary fat pad of female Balb/c mice as illustrated in Fig. 1A. When tumors reached 6–7 mm, 100 µL of 60% MRF was injected i.t (day 17) for treatment groups or 100 µl PBS in control group. One group was treated 24 hours after MRF injection with direct magnetic field application for 5 consecutive days (days 18–22). (A) Tumor volume during and after MRF/Magnet treatments. Data is representative of one of six experiments (n = 9 mice/group). (B) Total number of tumor cells by flow cytometry after 5 days of magnet treatment showing smaller tumor load after magnet treatment and (C) tumor CFUs of bone marrow. MRF/magnet treatments result in inhibition of growth of metastatic disease. (D) Representative flow staining for tubulin and 7AAD in the tumor. (E) Percentage of tubulin^+^7AAD^−^ (necrotic) cells in the tumor gated on CD45^−^ cells. (B–E) Data representative of one of two experiments with similar results (n = 3–4 mice/group). One-way or Two-way ANOVA. * P<0.05, ** P<0.01, *** P<0.001.

We next examined the effects of MRF/magnet application on growth kinetics of the primary tumor as well as metastatic progression. Mice that received MRF/magnetic field application exhibited significantly slower tumor growth in comparison to control groups (Fig. 2A). Overall lower tumor burden was observed after MRF/magnet treatments as depicted by the lower total cellularity of primary tumors (Fig. 2B). Injections of iron particles alone without magnetic field application did not impede to tumor progression. Primary tumor growth, while impeded by the combination treatment, was not completely abrogated as evident by the continual growth after cessation of treatment indicating that this approach alone is not sufficient in advanced tumor burdens. The 4T1 breast carcinoma model also spontaneously metastasizes after orthotopic primary tumor implantation [15]–[17]. Importantly, MRF/magnet treatment inhibited 4T1 metastasis within the bones after 5 days of magnet treatment in comparison to control groups (Fig. 2C). Thus, mechanical disruption of the primary tumor by MRF and magnetic field application results in anti-metastatic effects *in vivo*.

In order to determine if tumor inhibition by MRF/magnetic field application resulted in necrotic cell death and in immune activation, primary tumors were collected after 5 days of magnet application and stained for markers indicative of necrotic cell death by flow cytometry as determined by expression of tubulin [18]. The tubulin-positive cells that do not label with 7AAD represent cells that have damaged membranes and with degraded DNA and are considered necrotic [18], [19]. Following MRF/magnet therapy, total numbers of CD45(−) tumor cells were reduced, as would be expected with the delayed tumor growth (Fig. 2B). We observed that mice treated with MRF/magnet application demonstrated increased tumor necrotic (tubulin^+^7AAD^−^) cell death of CD45(−) cells (non-tumor cells or hematopoietic cells) (Fig. 2D–F) compared to MRF alone (Fig. 2D–E; 35% versus 50% respectively). We also determined if similar type of death occurred in the immune cells found in the primary tumor after MRF/magnet treatment. In contrast to what was observed in the CD45(−) tumor cells; there was no difference in the CD45(+) cells with regard to tubulin expression after MRF/magnet treatments in comparison to the other groups (Fig. S1A). Collectively, these findings demonstrate that *in situ* destruction of primary tumor via MRF/magnet treatment results in selective necrosis of malignant cells while preserving the tumor-infiltrating immune cells. Our model of tumor destruction by magnetic particles and its effects on immune activation is presented in Fig. S2.

### MRF and Magnetic Field Application of the Primary Tumor Results in Local Expansion and Activation of DCs

**Figure 3:**
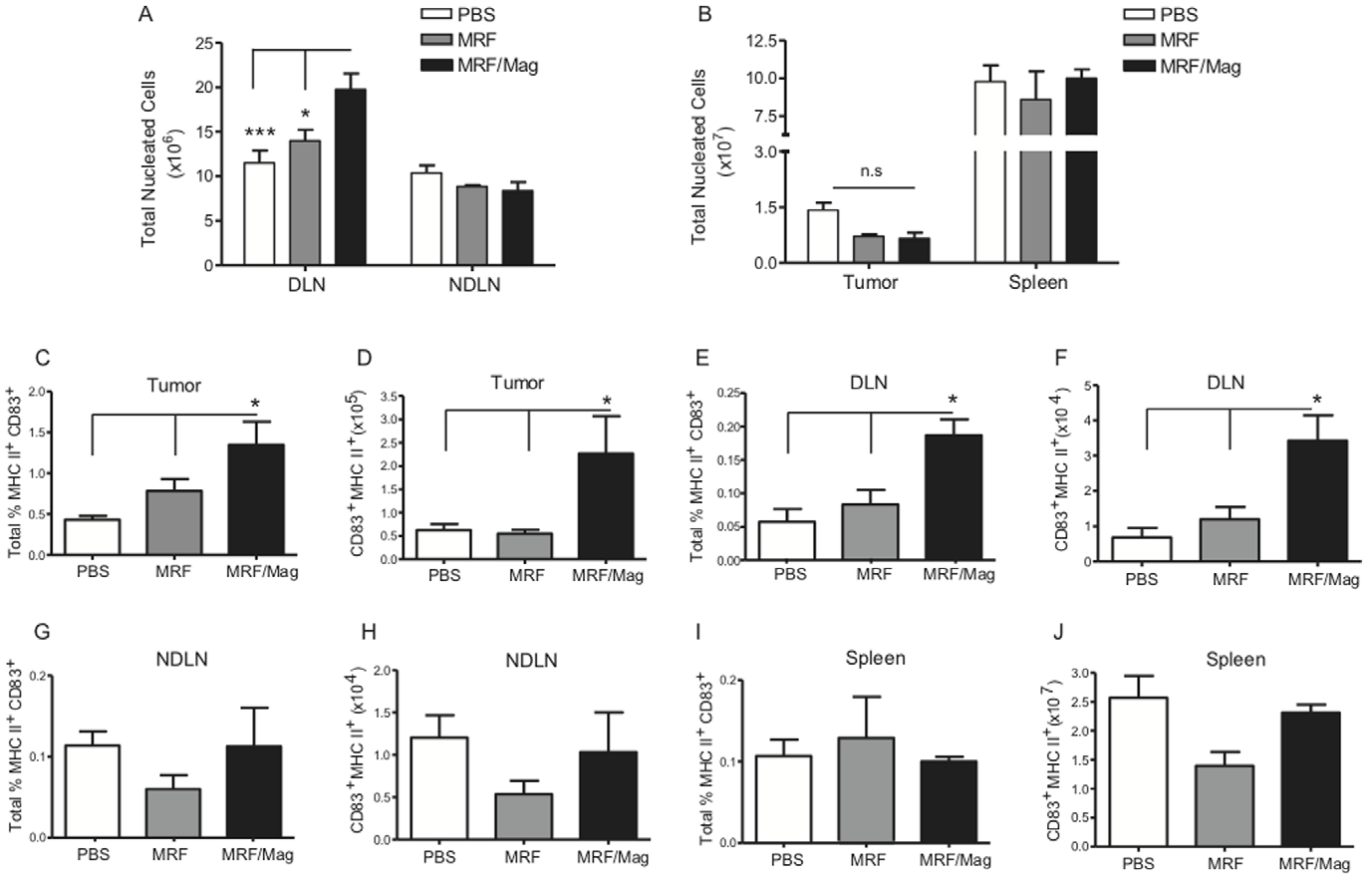
DC expansion and activation after MRF and magnetic field treatment. 4T1 tumors were injected into the mammary fat pad of female Balb/c mice as illustrated in Fig. 1A. Briefly, primary tumors were injected with 100 µL of 60% MRF i.t in the treatment groups, some mice received no further treatments and some mice received direct magnet application over the tumor for a total of 5 days. Control group received 100 µL PBS i.t. Five days after magnet treatments, DLN, NDLN, tumor and spleen were excised and analyzed by flow cytometry for (**A–B**) total number of nucleated cells in (**A**) DLN and NDLN or (**B**) tumor and spleen. All four organs were analyzed for CD83, MHC II expression gating on CD45^+^ CD11c^+^ CD19^−^ as follow: (**C–D**) represents both the total percentage and numbers of activated DCs in the tumor, (**E–F**) in the DLN, (**G–H**) in the NDLN and (**I–J**) in the spleen. Data (mean ± SEM) representative of one of four experiments (n = 3–4 mice/group). One-way ANOVA. * P<0.05, *** P<0.001. n.s: not significant.

We hypothesized that the mechanical disruption of the primary tumor by MRF/magnetic field application could activate antigen presenting cells (APCs) such as DCs. To determine whether MRF/magnetic field application could induce immune responses through local DC activation, mice received MRF/magnet application, MRF or PBS alone and we assessed the phenotype of the DCs at the local sites (tumor-draining-LNs [DLNs] and primary tumor) or peripherally (spleen and non-draining-LNs [NDLNs]). Only mice that received MRF/magnet application showed significantly increased cellularity in the tumor-DLN suggesting local recruitment of immune cells in comparison to the peripheral nodes/spleen (Fig. 3A–B). Cellularity of the primary tumor (Fig. 3B) is reduced compared to PBS treated group but not significant in comparison to either MRF or PBS alone groups which is consistent with the data presented in Fig. 2B at day 5 of magnet treatment. Additionally, we also observed a significant increase in the total percentage of mature DCs (CD11c^+^ MHCII^+^ CD83^+^) both within the tumor (Fig. 3C) and the DLNs (Fig. 3E) in comparison to groups receiving PBS or MRF alone. The combination of MRF/magnetic field application resulted in selective local immune activation as no effects on DCs were observed in distant NDLNs (Fig. 3G) and spleen (Fig. 3I). Increases in both percentage and numbers of DCs were observed (Fig. 3D and 3F) compared to systemic sites in the NDLNs and spleen (Fig. 3H and 3J), respectively. Thus, *in situ* tumor destruction results in the induction of antitumor immunity through local innate activation.

### MRF and Magnetic Treatments Increases Tumor-infiltrating Antigen-specific CD8(+) T Cells

**Figure 4:**
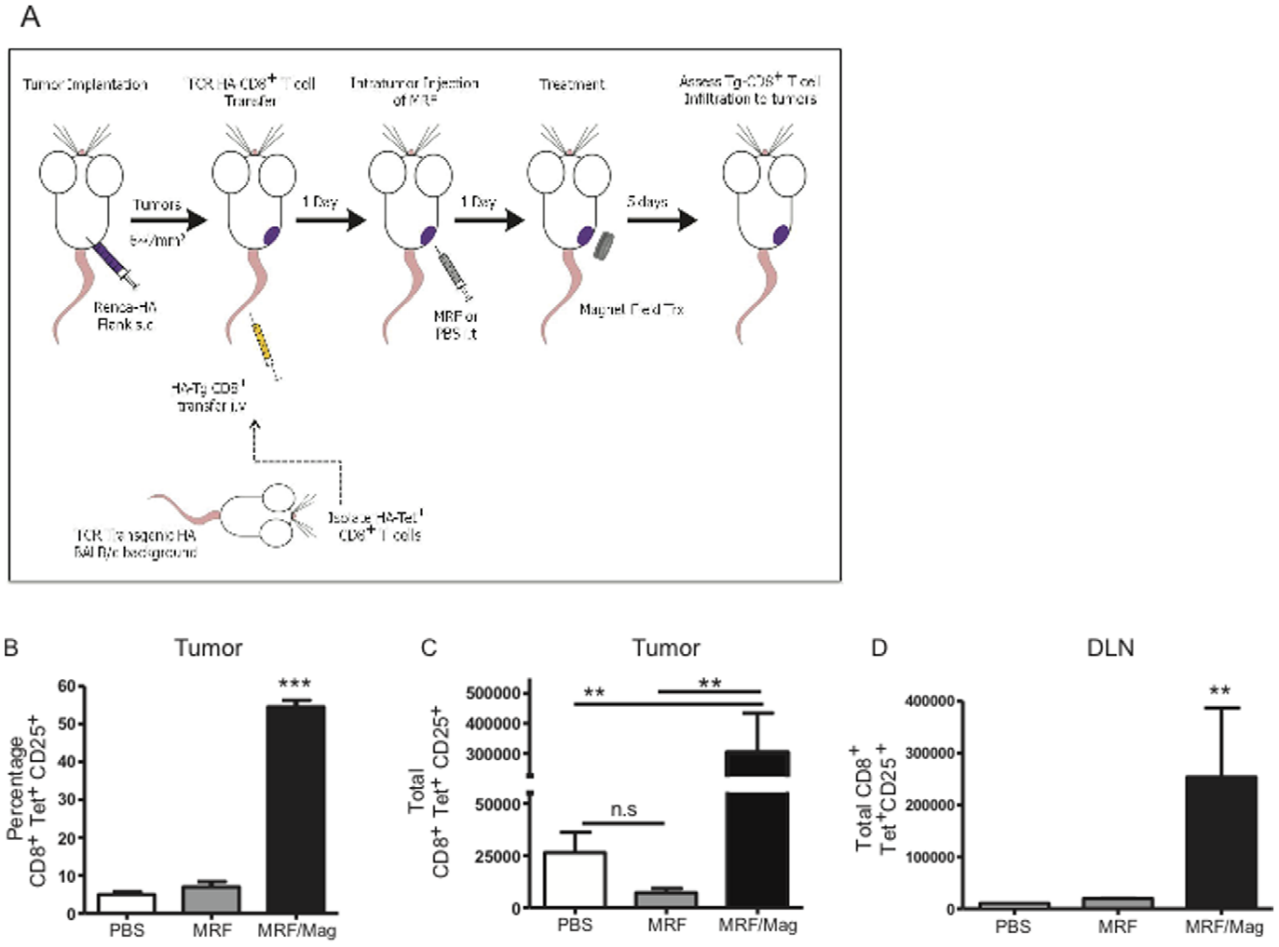
MRF and magnetic field application results in antigen-specific T cell accumulation. (**A**) Experimental model for antigen-specific responses following MRF and magnet treatments: BALB/c mice received Renca-HA on the right flank s.c. When tumors reached the desired size, mice first received 2×10^6^ CD8(+)-HA-Tet^+^ cells i.v. One day following Tg-CD8(+) T cell transfer, mice were administered MRF or PBS intratumor. 24 h later, some groups are left untreated or receive magnetic treatments for 5 min a day for 5 consecutive days. After 5 days of daily magnetic field treatments, tumors or tumor-DLNs were collected and assayed by flow cytometry. (**A**) Percentage and (**B**) total number of transgenic HA-Tg-CD8(+)-CD25+ T cells in Renca-HA tumor. (**C**) DLNs from the same mice in (**A–B**) were analyzed for homing of HA-Tg-CD8(+)CD25+ T cells. Data representative of one single experiment (n = 3 mice/group in PBS or MRF group and n = 5 mice in the MRF/magnet group). One-way ANOVA. ** P<0.01, *** P<0.001, n.s: not significant.

We next investigated the CD8(+) T cell responses after MRF/magnetic field treatments and determined whether increased antigen-specific tumor responses resulted. To address this, BALB/c mice were injected with a Renca cell line transfected with the influenza virus hemagglutinin (HA) (Renca-HA) on the right flank s.c as illustrated in Fig. 4A. When tumors reached the desired size, T cells from HA-specific CD8(+) transgenic (HA-Tg-CD8(+)) mice were isolated. HA-Tg-CD8(+) T cells were adoptively transferred into Renca-HA bearing mice. HA-Tg-CD8(+) were isolated by magnetic bead isolation for CD8(+) T cells and purity was checked by flow cytometry staining with a tetramer specific for the HA peptide. At the end of MRF/magnet treatments (day 5 of magnet or equivalent to day 7 after HA-Tg-CD8(+) T cell transfer), we determined the fraction of activated tumor-specific CD8(+) T cells homing to the tumor by evaluating for CD8(+)-tetramer-HA^+^-CD25^+^ T cells using flow cytometry. As depicted in Fig. 4B, MRF/magnet application induced a significant increase in the percentage of CD8(+)-tetramer^+^-CD25^+^ T cells to the Renca-HA-MRF/magnet-treated tumor, and not in control groups (Fig. 4B) with approximately 13-fold increase in overall total numbers of HA-Tg-CD8(+) T cells homing to the primary tumor (Fig. 4C). We observed a similar increase of antigen-specific CD8(+) T cells homing to MRF/magnet-treated Renca-HA tumor-DLNs as well, with approximately 20-fold increase in total number of HA-Tg-CD8(+) T cells in the DLNs as compared to either MRF or PBS groups (Fig. 4D). These data demonstrate that antigen-specific CD8(+) T cell recruitment occurs to primary tumors after MRF/magnetic field application.

### Increased Immune Responses and Anti-metastatic Effects Following MRF/Magnetic Field Application in Comparison to Cryoablation

Tumor ablative therapies, such as cryoablation or radiofrequency ablation (RFA), have been successfully used in the clinical settings for destruction of primary tumors [1], [2], [5], [7], [9]. RFA has shown some anti-tumor responses in human breast cancer and some mouse tumor models with modest success [20], [21]. Nevertheless there are no reports on the efficacy of RFA in disseminated disease. Cryosurgery of primary tumor in mice has demonstrated accumulation of CD80^+^ CD11c^+^ DCs in the LNs as well as induction of IFN-γ^+^ CD8(+) T cells in the tumor-DLN but only when cryosurgery was combined with intratumoral CpG administration [22].

**Figure 5:**
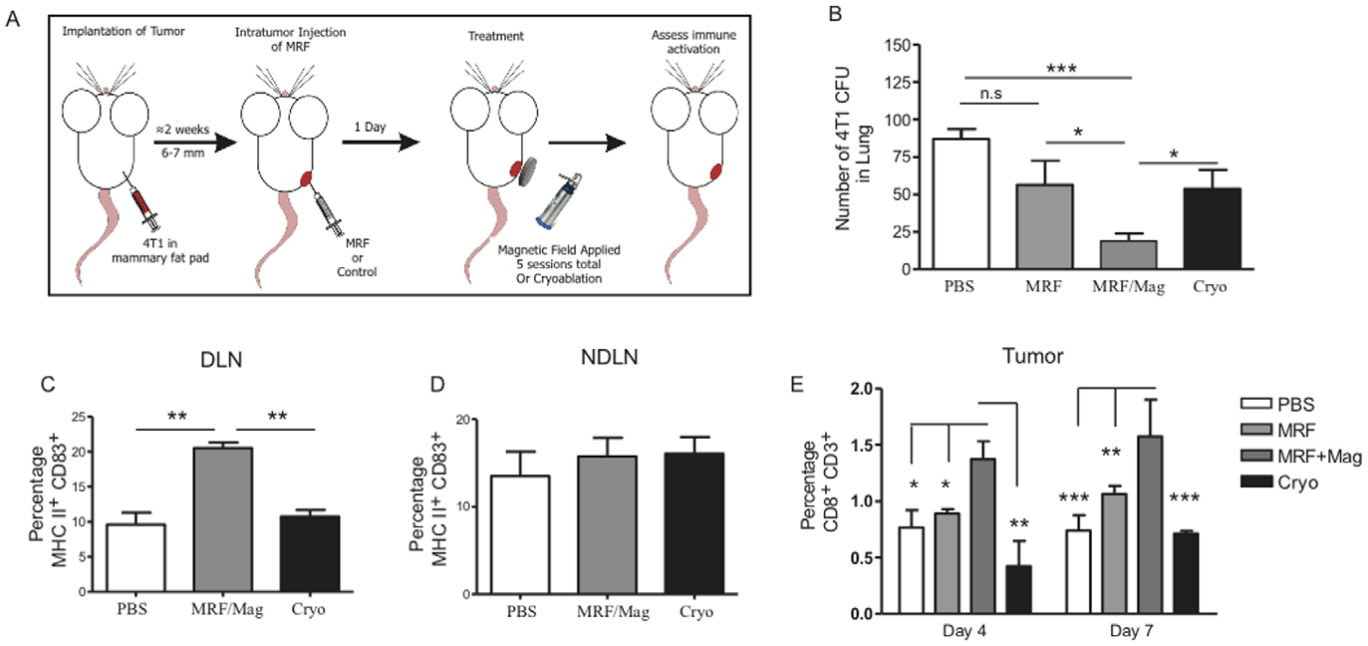
Augmented immune activation and inhibition of metastasis with MRF/magnetic treatments in comparison to cryoablation. (A) Experimental design of MRF and magnet treatments and cryoablation. BALB/c mice received 4T1 orthotopically on one side of the mammary fat pad. When tumors reached 6–7 mm in size, some mice received one of the following: PBS i.t, MRF i.t, MRF i.t and magnet treatment (5 min/day for 5 days) or cryoablation once. (B) Lung cells were collected and plated for tumor CFUs (after 4 days of magnet application). (C–D) Percentage of activated DCs (MHC II^+^ CD83^+^ of CD45^+^ CD11c^+^ CD19^−^) in the DLNs (B) and NDLNs (C). (E) Percentage of CD8(+) T cells (gated on CD3+ CD45+) in primary tumor in each group relative to 4 or 7 days of magnet treatments. Values represent the mean ± SEM of one of two experiments with similar results (n = 3–5 mice/group). One-way or Two-way ANOVA. * P<0.05, ** P<0.01, *** P<0.001, n.s: not significant.

We next compared the *in situ* tumor destruction approach using MRF/magnetic field application to cryoablation and its consequences on metastatic disease and immune function. In this model, mice received PBS, MRF or MRF/magnet, or cryoablation by freezing and thawing the primary tumor site (Fig. 5A). While both therapies resulted in *in situ* tumor death, only MRF/magnet treatment resulted in delayed metastatic outgrowth in the lung (Fig. 5B). Additionally, only MRF/magnet treatment resulted in significant increases in the frequency of mature DCs (CD11c^+^MHC II^+^CD83^+^) in the tumor-DLNs only and not the NDLNs (Fig. 5C–D) in comparison to mice receiving cryoablation. We also observed a significant increase in the percentage of CD3+CD8(+) T cells infiltrating the primary tumor after magnetic application but not with cryoablation (Fig. 5E). Our findings are in agreement with other reports that have demonstrated the need to use cryoablation in combination with other immunomodulatory agents in order to obtain demonstrable immune modulation [22], [23]. In contrast, MRF/magnet without addition of an immunomodulatory therapy induced enhanced anti-tumor responses with increased tumor-infiltrating CD8(+) T cells and inhibition of outgrowth of metastatic breast cancer. Collectively, these experiments demonstrate that *in situ* tumor injury via MRF/magnet application can activate DCs, recruit them to primary tumor site, activate CD8(+) T cells, and initiate anti-tumor effects to distant sites.

### Combination of MRF/Magnetic Field Application with Suboptimal Systemic Immunotherapy Leads to Further Increases in Systemic Anti-tumor Responses

**Figure 6:**
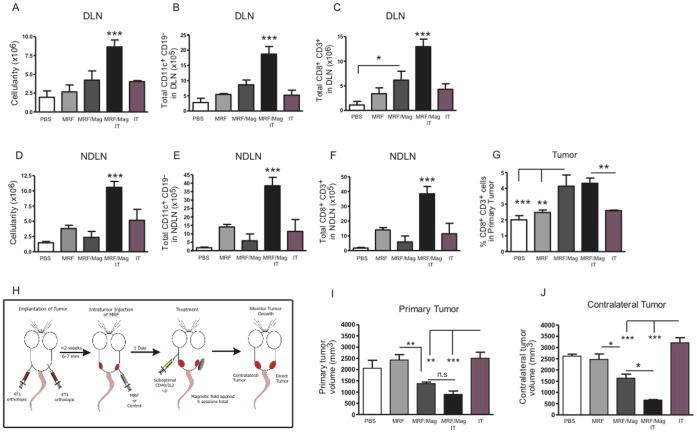
Combination of MRF/magnetic field application with immunotherapy results in heightened systemic anti-tumor responses. 4TI breast cancer cells were implanted into the mammary fat pad of BALB/c mice. Treatment was initiated when tumors reached an average volume of 6–7 mm. Mice received one of the following 5 treatments: 1) PBS i.t, 2) MRF i.t alone, 3) MRF and magnet, 4) MRF+ magnet + anti-CD40 (25 ug) and recombinant human IL2 (2.5 ×10^∧^5 IU) i.p or 5) anti-CD40 and IL2 alone. Anti-CD40 was given for 5 consecutive days starting the same day as the magnetic field treatments and IL2 was administered on days 2, 5, 9 and 11 post MRF injections. (A–G) Immune effects of combination of low dose anti-CD40/IL2 with MRF/magnet after 5 days of magnet treatment and anti-CD40/IL2. (A, D) Total nucleated cells in the DLN and NDLNs, respectively. (B) Total percentage of DCs (CD11c^+^ CD19^−^ CD45^+^) in DLNs and (E) NDLNs. (C) Total CD3+ CD8(+) T cells in DLNs or (F) NDLNs or (G) in the tumor. Data representative of one of three experiments with similar results (n = 3–4 mice/group). (H) Experimental model for systemic anti-tumor effects: 4TI breast cancer cells were implanted s.c. on the right and left side of the mammary fat pad of BALB/c mice. Only the right tumors were injected with MRF or PBS. Some groups were further treated for 5 consecutive days with magnetic field. Other groups received anti-CD40 (25 ug) on the same days of magnet treatment and rh-IL2 (2.5×10^5^ IU) (days 2, 5, 9 and 11 post MRF injections). (I-J) Tumor volumes after 28 days post tumor inoculation or equivalent to 12 days after start of magnet treatments are represented for (J) primary tumors that received MRF or (J) the contralateral untreated tumors. Data representative of one of five experiments with similar results (n = 5–7 mice/group). One-way ANOVA. * P<0.05, ** P<0.01, *** P<0.001. n.s: not significant. IT: anti-CD40/IL2.

We have previously shown that immunotherapy using agonist CD40 antibodies combined with high doses recombinant human IL-2 results in synergistic anti-tumor responses against metastatic disease in tumor bearing mice [24]. Therefore, we combined MRF/magnet treatment with anti-CD40/IL2 to treat disseminated disease and determine if greater anti-tumor responses and systemic immune activation can be achieved with this multi-approach therapy. Mice received 4T1 in the mammary fat pad and followed by PBS or MRF intatumor injections with or without magnetic field treatments. We also added two groups that received agonistic anti-CD40 and IL/2. In combination with magnet treatments one group received systemic low doses of anti-CD40/IL2 intraperitoneal. An additional group received the immunotherapy alone without MRF. This lower dose of anti-CD40/IL2 resulted in no overt toxicities otherwise seen with high doses [25]–[30]. The combination of MRF/magnetic treatments with low dose immunotherapy resulted in significant increases in total DLN and NDLN cellularity (Fig. 6A and 6D) as well as expansion of DCs to both the DLNs and NDLNs (Fig. 6B and 6E). Thus, the addition of immunotherapy allowed for a systemic expansion of DCs when combined with MRF/magnetic field application.

MRF and magnetic field application with or without immunotherapy in tumor-bearing mice resulted in increased frequencies of CD8(+) T cells within the primary tumor (Fig. 6G). This local expansion of CD8(+) T cells is further supported by the increase in CD8(+) T cell numbers to the tumor-DLN (Fig. 6C). MRF/magnet alone did not result in significant CD8(+) T cell expansion at peripheral LN (NDLNs) (Fig. 6F), but this did occur when it was combined with anti-CD40/IL2 (Fig. 6F).

Because we observed increased local and systemic immune activation with this multi-approach therapy, we further examined the effects of this combinatory treatment on growth of disseminated tumor. To address this, we used the same treatments groups as stated above except this time mice received 4T1 in the mammary fat pad on both sides (Fig. 6H). Only the ispilateral-tumor (primary) received i.t injection of MRF or PBS. One day following MRF or PBS implantation, magnet treatments were initiated on the ispilateral-tumor for a total of 5 treatments. In combination with magnet treatments, one group received systemic low doses of anti-CD40/IL2. An additional group received anti-CD40/IL2 alone (Fig. 6H) and volumes of the primary and contralateral tumors were assessed. Our data shows that MRF/magnetic field application resulted in inhibition of growth of the contralateral tumor, which was left untreated (Fig. 6J), indicating induction of systemic anti-tumor immunity. Most importantly, combination of MRF/magnetic field and anti-CD40/IL2 resulted in significantly greater inhibition of both primary (Fig. 6I) and contralateral-tumor growth (Fig. 6J). These findings indicate that even greater disseminated anti-tumor effects can be obtained using MRF/magnetic field application as an adjuvant with immunotherapy.

## Discussion

A major hurdle in generating successful anti-tumor responses is associated with the fact that the vast majority of cancer treatments involve either removal of the tumor antigen load (surgery) which can be crucial for eliciting anti-tumor responses or involve the use of cytoreductive therapies (chemotherapy and radiation) which can suppress the immune responses. Furthermore, the tumor microenvironment itself is immunosuppressive and with the weakly immunogeneic nature of cancers this results in lack of sufficient “danger” signals to activate immune responses. Thus, means to induce destruction of the tumor while preserving the potentially diverse antigen pool as well as induction of “danger” signals to activate DCs may allow for more vigorous anti-tumor systemic responses to metastatic disease (Figure S2 illustrates our model of tumor death and immune activation).

In this study, we developed a novel means of mechanical destruction of the tumor *in situ*, without the use of cytoreductive agents. We observed that injection of MRF comprised of iron particles at the site of the tumor followed by application of a magnetic field led to immediate consolidation of the particles resulting in tumor destruction architecture and induced necrotic tumor death, while preserving the tumor-infiltrating immune cells. However, the exact mechanism of tumor necrosis is still unclear. It is possible that the immediate coalescence of the iron particles upon magnetic treatment, leads to disruption of the tumor microvascular endothelial and stromal structure that holds the tumor cells together. This in turn causes a loss of the tumor extracellular matrix and tumor blood vessels necessary for the proper supply of nutrients, soluble factors and oxygen necessary for the support of tumor growth and metastasis [31], [32].

This *in situ* primary tumor destruction using MRF/magnetic field was capable of inducing necrotic death of the primary tumor, maturation and accumulation of DCs within the tumor and the tumor-DLNs as well as inhibition of tumor growth in comparison to control groups. In these studies, the primary tumor, while showing inhibition, still progressed with MRF/magnet application. Notably, the majority of the treated tumor with MRF and magnetic field was often necrotic and had ulceration necessitating euthanization of the recipients. The orthotopic breast cancer tumor 4T1 is a highly aggressive tumor that metastasizes as early as 7 days post tumor implantation depending on concentration of tumor injected [14]. Traditionally, 4T1 tumors are surgically removed about two weeks post inoculation and this has been shown to result in survival up to 150 days in 60–80% of the mice [33], [34]. In our model, no long term survival were established as the 4T1 tumors were not resected due to the necessity to keep the tumor mass in order to inject the iron particles intratumor. By giving a high amount of tumor cells (2×10^∧^5 4T1) and not resecting the primary tumor, we have a very fast growing and aggressive tumor that did not allow us to establish survival studies. In the present study, MRF/magnetic field application directly on primary tumor has shown greater and significant ability to activate and expand DCs within the tumor-DLNs as well as induce CD8(+) T cell homing to the primary tumor in comparison to tumors undergoing cryoablation. The failure of cryotherapy to induce comparable immune activation and to inhibit metastasis [3], [35], [36] is consistent with other reports showing that generation of immune activation after cryotherapy necessitates combination with other agents (CpG, IL-7) [21], [23]. Thus, mechanical destruction by MRF/magnetic field application is sufficient to induce enough tumor death while possibly sparing immune cells. However, the preservation of the immune cells from death cannot be ascertained due to the dynamic process of influx of immune cells to tumor site.

Cancer cells are known to evolve means to escape the immune system and induce a suppressive microenvironment [37], [38]. During tumor progression, a plethora of immune cells infiltrate the tumor site, however some of these cells aid in tumor sculpting and limit prolonged immune responses [39], [40]. Tumor associated macrophages (TAMs), T regulatory cells, granulocytes, plasmacytoid DCs, and immature DCs (iDCs) have all been implicated in tumor invasion and immune tolerance [41]–[44]. Additionally, other suppressive factors found in the tumor microenvironment including interleukin-10 (IL-10), transforming growth factor β, prostaglandine E2 (PGE2), and indoleamine 2,3-dioxygenase (IDO), lead to T cell suppression [45]–[50]. In fact, several studies have demonstrated that iDCs inhibit effector T cells by secreting vascular endothelial growth factor (VEGF), IL-10, IDO and arginase 1 (ARG1) among other factors [42], [51]–[55]. Furthermore, myeloid derived suppressor cells (MDSCs) are highly expressed by 4T1 tumor bearing mice [56], [57] among other tumor models [58]–[60]. Ostrand-Rosenberg *et al*. have demonstrated in a 4T1 model that MDSCs secrete IL-10 and decrease IL-12 production by macrophages [61], [62]. The presence of both MDSCs and IL-13 induced type 2 macrophages has also been demonstrated to inhibit T cell function in 4T1 tumor bearing mice [63]. In addition, MDSCs express receptors to PGE2 and that blockade of these receptors results in a reduction of MDSCs accumulation to 4T1 tumors and delays tumor growth [45]. Inhibition of MDSCs function in mice can indeed reverse immune tolerance and inhibit tumor growth [39], [64]. Collectively, all the above described suppressive cells and factors play a role in 4T1 tumor escape.

In our studies, while modest direct anti-tumor effects were obtained using this approach, combination of MRF/magnetic field treatment with immunotherapy using suboptimal doses of agonistic anti-CD40/IL2 resulted in significantly greater and, importantly, systemic anti-tumor responses. Thus, injury to the tumor while preserving the antigen pool and immune cell responses may allow for more vigorous anti-tumor responses to metastatic disease without the need for cytoreductive therapies. This adjuvant approach also allows for far less amounts of immunotherapy to be applied. It is likely that local administration of immunotherapy by anti-CD40 or CpG will result in even greater effects with lesser toxicities.

Overall, this novel means of *in situ* destruction of murine breast tumor by mechanical disruption of tumor architecture via local injection of magnetic particles shows promising anti-tumor responses by activation of both innate and adaptive immunity. This can be readily applied clinically in which primary breast or skin tumors are first treated with iron particles and magnetic field application to allow for local immune activation, which can be amplified with use of adjuvants or immunotherapy. This regimen provides a minimally invasive means in which primary tumor is not removed but instead targeted from within, allowing for tumor antigen dispersal, immune cell recruitment, antigen uptake and activation of innate and adaptive immune effectors critical for anti-tumor responses, while at the same time permits slower primary tumor growth. Surgical resection of the primary tumor can then occur. This would then allow for better control and eradication of disseminated disease when combined with immunotherapy and possibly at lower doses allowing for less toxicities often observed with administration of systemic high dose immunotherapy.

## Materials and Methods

### Animals

Female BALB/c mice, age 8–12 weeks old were purchased from Charles River or the National Cancer Institute (NCI, Frederick, MD). CBy.Cg-Thy1^a^Tg are Clone 4 Vβ8.2/Vα10 TCR-transgenic (Tg) mice (hemagglutinin protein Tg [HA-Tg]) that were backcrossed for ten generations to BALB/cBy prior to crossing to BALB/c-Thy1^a^. The CD8(+) T cells from HA-Tg mice express TCRαβ chains specific for the IYSTVASSL peptide Ag, encoded by amino acid residues 518–528 of the PR8 virus HA protein, and presented in the context of H-2K^d^ [65]–[67]. HA-Tg mice were purchased from Jackson Laboratory (Bar Harbor, ME). All of the animal experiments were approved by the Institutional Animal Care and Use Committee of the University of Nevada Reno and the University of California Davis.

### Tumor Cell Lines and Reagents

4T1 metastatic mammary carcinoma cell line was obtained from Dr. Kenneth Hunter at the University of Nevada Reno. Renca-HA (Renca tumor transfected with the hemagglutinin peptide [HA]) was obtained from Dr. Thomas J. Sayers (NCI Frederick, MD) [68]. Cells were cultured RPMI1640 medium containing FBS, penicillin, streptomycin, glutamine, non-essential amino acids, sodium pyruvate, Hepes and 2-mercaptoethanol (2-Me) at 37°C in a humidified atmosphere containing 5% CO_2._ Cell viability was checked by trypan blue and counted with a hemacytometer. Cultures of Renca-HA were supplemented with G418 (1 mg/ml) sulfate solution (Invitrogen, Carlsbad, CA). 2×10^5^ 4T1 cells were suspended in 0.1 ml PBS and injected orthotopically into the mammary fat pad. 2×10^6^ Renca-HA cells were injected in a 0.1 ml volume s.c into the right flank of BALB/c mice.

Agonistic anti-mouse CD40 antibody (clone FGK115B3) was generated via ascites production in our laboratory, as previously described [24]. Recombinant human interleukin-2 (IL-2; TECIN Teceleukin) was provided by the NCI (Frederick, MD). BALB/c mice received low dose anti-CD40/IL-2 as specified in Fig. 6.

### Magnetorheological Fluid (MRF) and Magnets

MRF is composed of surface modified iron particles from carbonyl iron powder (6–8 microns) (BASF, USA) commercially available. The procedure for surface coating of iron particles using various polymers via atom transfer radical polymerization (ATRP) were described in [69]. For magnetic field treatments, permanent magnets with a magnetic field of 0.4 Tesla were used. The magnets dimensions are: 1.1 cm for the diameter and 0.8 cm for the height.

### Tumor Models for MRF and Magnet Treatments

#### Model for direct anti-tumor effects (primary tumor)

2×10^5^ 4T1 in 0.1 ml PBS were injected orthotopically into the mammary fat pad. Tumor size was measured with a Vernier caliper. When tumor reached 6–7 mm in dimension, mice were randomized between three different groups with 5–9 mice per group for tumor measurements and 3–5 mice per group for histology or flow cytomerty analysis. The three treatment groups are as follow: One group received 0.1 ml PBS intratumor (i.t), the second group received 60% w/v MRF in 0.1 ml PBS i.t without further treatments and the third group received 60% w/v MRF in 0.1 ml PBS i.t followed by magnetic field treatment. Specifically, one day after MRF injections, mice in group three were treated with magnets by direct application over the primary tumor. Mice in group three received one session a day of magnetic field treatments for 5 min for 5 consecutive days, the number of mice in each experiments is specified in the figure legends. Tumor measurements were recorded everyday during magnet application and every other day after cessation of magnet treatments. Perpendicular dimensions of the tumor were measured such as a = length and b = width and tumor volumes were calculated by *a*×*b*×*c*, with *c* = to the smallest dimension from *a* or *b*. Refer to Fig. 1A.

#### Model for antigen specific responses

Refer to Fig. 4A.

#### Model for systemic anti-tumor effects (contralateral model)

Refer to Fig. 6H.

### Cryoablation

Mice were placed in a sanitized laminar hood and anesthetized i.p with Nembutal (6 µg/g) prior to cryoablation. Tumors were wiped with alcohol then frozen using liquid nitrogen cryoguns (Brymill Cry-AC) by direct application to primary 4T1 tumors. Mice received a total of 2 treatment cycles, 20 seconds each of freezing and thawing of the tumor, allowing the tumor to thaw passively and then freezing the tumor a second time. After completion of cryoablation, mice were placed under a warming lamp during the recovery period of the mice to avoid hypothermia and were monitored closely until they recover. Cryoablation was completed on the day where other treatment groups received magnetic field treatments. Refer to Fig. 5A.

### Cell Preparation

Spleen, tumor-draining-LNs (DLNs), non-draining-LNs (NDLN), primary and contralateral tumors were brought to single-cell suspensions with prior digestion with collagenase type IV at 37°C on a shaker for 45–60 min. Cells were further broken down, filtered and washed in PBS containing 5% FBS. Spleen suspensions were lysed of red blood cells by incubation (5–10 min) with tris buffer containing ammonium chloride (pH = 7.2), washed, counted on a Coulter Z1 cell counter (Coulter Electronics) and adjusted for flow cytometry.

### Purification of Transgenic CD8(+) T Cells

Spleen and LNs of naïve clone 4 transgenic mice were brought into single-cell suspension, pooled together and CD8(+) T cells were enriched by negative selection using EasySep enrichment kit (STEMCELL Technologies). First, cells were incubated with a cocktail of antibodies against CD11b, CD19, CD45R, CD49b and TER119 (STEMCELL Technologies) for 15 min at 4°C and then with biotinylated antibodies cocktail against the above unwanted surface antigens for 15 min at 4°C. Magnetic nanoparticles were then add to the cells, incubated at 4°C then placed on the EasySep® Magnet and the desired fraction of CD8(+) was poured off while tube still on the magnet. Magnet isolation was repeated twice. Purity of HA-Tg-CD8(+) T cells was verified before intravenous transfer into syngeneic BALB/c mice using an H-2K^d^ flu HA tetramer recognizing AA_240–248_ peptide (IYSTVASSL). APC-Tetramer was made upon request at the NIH Tetramer Facility, Emory University, Atlanta, GA. Refer to Fig. 4A for experimental model.

### Flow Cytometry

10^6^ single-cell suspensions were labeled with Fc block then incubated with anti-mouse antibodies including the following: CD45, CD19, CD11c, MHC II, CD83, Tubulin, CD3e, CD8, CD25, APC-hemagglutinin-Tetramer (NIH Tetramer Facility, Emory University, Atlanta, GA) and 7AAD (BD Pharmingen). Listmode data files were collected on a LSRII or a LSRFortessa cell analyzer using the FACSDiva® software (Becton Dickinson, San Jose, CA) or on a S1400 4-laser with the Cellcapture software (Stratedigm, San Jose, CA). All data sets were analyzed using FlowJo software (TreeStar).

### CFU Assay

Lung cells were digested with collagenase IV (at 2 mg/ml in a 1 to1 ratio with FBS) for 45 min at 37°C on a shaker, processed a second time, washed in PBS containing 5% FBS, brought to suspension in culture media, counted on a Coulter counter and adjusted to 1×10^7^ cells/ml. For bone marrow (BM) colony-forming units (CFUs), BM cells were flushed with sterile PBS, washed, brought up to single suspension, counted and adjusted to 3×10^6^ cells/ml. For CFU assay, lung cells or BMCs were cultured in 35-mm petri dishes in colony assay medium containing methylcellulose, FBS, IMDM, penicillin, streptomycin, and 2-Me without any growth factors. Cultures were established in triplicate for each animal and maintained for 2 weeks at 37°C. Tumor colonies were counted on a stereo microscope (Nikon, Melville, NY). One colony represents >50 tumor cells.

### Histology

Primary tumors were excised at the end of MRF treatment, fixed in 10% paraformaldehyde, embedded in paraffin, cut in sections and stained with H&E at the Histology Consultation Services in Everson, WA. Images were captured with an Olympus BX4 microscope equipped with a Q-color3 camera and 10x numerical aperture objective lens.

### *In vivo* Imaging

Mice were anesthetized with i.p injections with Nembutal at 6 µg/g, then placed in the prone position and scanned on a Phillips Brilliance Big Bore CT scanner using 1 mm axial cuts. Images were analyzed using Pinnacle software. Axial cuts and sagittal and coronal reconstructions for each animal were reviewed in detail by a board certified Radiation Oncologist at the University of California Davis. Two animals per treatment group were scanned at days 2 and 14 post i.t MRF injections. Immediately after imaging mice were placed under a warming lamp for recovery to avoid hypothermia and watched closely until they woke up then put back in their cages until the next imaging session. Results shown are representative cuts from one animal/group at each time point.

### Statistics

Statistical analysis (mean ± SEM) was performed using Prism software (GraphPad Software Inc.) For analysis of 3 or more groups, the non-parametric ANOVA test was performed with the Bonferroni post-test. Analysis of differences between 2 normally distributed test groups was performed using the Student’s *t*-test with Welch’s correction. * P<0.05, ** P<0.01, *** P<0.001.

## Supporting Information

Figure S1 **Comparable death of CD45^+^ hematopoietic cells within primary tumors following MRF and magnet treatments. (S1A):** 4T1 tumors were established and treated as in Fig. 1A. Briefly, when tumors reached 6–7 mm, mice were randomized between 3 different groups. One group received 100 µl PBS i.t, a second group received 100 µl of 60% MRF w/v in PBS i.t without further treatments. A third groups received 60% MRF followed by magnetic field treatment for 5 min/session for 5 consecutive days. After 5 days of magnet application, tumors were collected and analyzed for death of CD45^+^ cells in the primary tumor. Percentage of necrotic (tubulin^+^7AAD^−^) non-tumor cells (CD45^+^ cells) in the primary tumor after PBS, MRF, and MRF/magnetic field treatments. Two- way ANOVA based on Bonferroni post tests was performed to determine significance. n.s: not significant.
(TIFF)

Figure S2 **Schedule of MRF/magnetic field treatments, tumor death and immune activation.** 4T1 cells are injected into the mammary fat pad of female BALB/c mice. When tumors reached 6–7 mm, 100 µl of 60% MRF w/v in PBS is injected into the tumor for treatment groups or 100 µl PBS in control group. One group receives magnetic field treatments by direct application of permanent magnets on the primary tumor. Mice receive 5 min/session of magnet treatments using a 0.4 Tesla magnet starting 24 hours after MRF injection for 5 consecutive days. Another group receives MRF i.t and no further treatments. Magnet treatments lead to aggregation of the iron particles, tumor death by necrosis, release of tumor antigen, recruitment of DCs and CD8(+) T cells.
(TIFF)
