# Supplementary figures and images for: Mechanical Disruption of Tumors by Iron Particles and Magnetic Field Application Results in Increased Anti-Tumor Immune Responses
Source: PLoS One. 2012 Oct 25;7(10):e48049. doi: 10.1371/journal.pone.0048049 (PMC3485005; doi:10.1371/journal.pone.0048049)

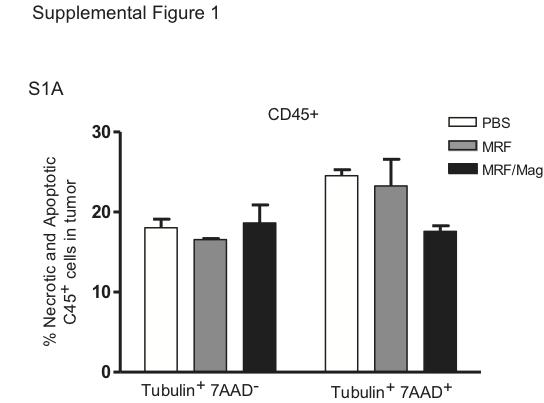

Supplement: Figure S1 — Comparable death of CD45+ hematopoietic cells within primary tumors following MRF and magnet treatments. (S1A): 4T1 tumors were established and treated as in Fig. 1A. Briefly, when tumors reached 6–7 mm, mice were randomized between 3 different groups. One group received 100 µl PBS i.t, a second group received 100 µl of 60% MRF w/v in PBS i.t without further treatments. A third groups received 60% MRF followed by magnetic field treatment for 5 min/session for 5 consecutive days. After 5 days of magnet application, tumors were collected and analyzed for death of CD45+ cells in the primary tumor. Percentage of necrotic (tubulin+7AAD−) non-tumor cells (CD45+ cells) in the primary tumor after PBS, MRF, and MRF/magnetic field treatments. Two- way ANOVA based on Bonferroni post tests was performed to determine significance. n.s: not significant. (TIFF) [file pone.0048049.s001.tiff]

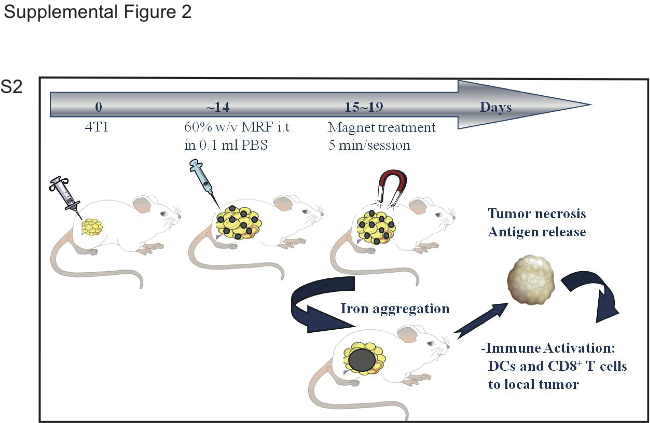

Supplement: Figure S2 — Schedule of MRF/magnetic field treatments, tumor death and immune activation. 4T1 cells are injected into the mammary fat pad of female BALB/c mice. When tumors reached 6–7 mm, 100 µl of 60% MRF w/v in PBS is injected into the tumor for treatment groups or 100 µl PBS in control group. One group receives magnetic field treatments by direct application of permanent magnets on the primary tumor. Mice receive 5 min/session of magnet treatments using a 0.4 Tesla magnet starting 24 hours after MRF injection for 5 consecutive days. Another group receives MRF i.t and no further treatments. Magnet treatments lead to aggregation of the iron particles, tumor death by necrosis, release of tumor antigen, recruitment of DCs and CD8(+) T cells. (TIFF) [file pone.0048049.s002.tiff]
